# Unmanned Airborne Target Detection Method with Multi-Branch Convolution and Attention-Improved C2F Module

**DOI:** 10.3390/s25196023

**Published:** 2025-10-01

**Authors:** Fangyuan Qin, Weiwei Tang, Haishan Tian, Yuyu Chen

**Affiliations:** School of Physics and Electronics, Hunan Normal University, Changsha 410081, China; 202220112382@hunnu.edu.cn (F.Q.); haishan_tian@163.com (H.T.); 17803602130@163.com (Y.C.)

**Keywords:** small-target detection, multi-branch convolution, improved layer C2F module, attention mechanism

## Abstract

In this paper, a target detection network algorithm based on a multi-branch convolution and attention improvement Cross-Stage Partial-Fusion Bottleneck with Two Convolutions (C2F) module is proposed for the difficult task of detecting small targets in unmanned aerial vehicles. A C2F module method consisting of fusing partial convolutional (PConv) layers was designed to improve the speed and efficiency of extracting features, and a method consisting of combining multi-scale feature fusion with a channel space attention mechanism was applied in the neck network. An FA-Block module was designed to improve feature fusion and attention to small targets’ features; this design increases the size of the miniscule target layer, allowing richer feature information about the small targets to be retained. Finally, the lightweight up-sampling operator Content-Aware ReAssembly of Features was used to replace the original up-sampling method to expand the network’s sensory field. Experimental tests were conducted on a self-complied mountain pedestrian dataset and the public VisDrone dataset. Compared with the base algorithm, the improved algorithm improved the *mAP*50, *mAP*50-95, *P*-value, and *R*-value by 2.8%, 3.5%, 2.3%, and 0.2%, respectively, on the Mountain Pedestrian dataset and the *mAP*50, *mAP*50-95, *P*-value, and *R*-value by 9.2%, 6.4%, 7.7%, and 7.6%, respectively, on the VisDrone dataset.

## 1. Introduction

With the rapid development of unmanned aerial vehicle (UAV) technology, the application of UAVs in emergency rescue [1] and ecological protection [2] is becoming increasingly widespread. Traditional search-and-rescue methods are often limited by problems such as complex terrain, insufficient manpower, and slow response times, which make it difficult to meet the demand for efficient rescue. However, through the advantageous combination of UAV technology with target detection algorithms, new solutions for mountain searches have been found. With advantages such as flexibility, mobility, and wide coverage, drones can quickly access difficult-to-reach areas. Additionally, by integrating advanced target detection technology, drones can analyze image and video data in real time to efficiently locate missing people.

However, the widespread application of this technology is still hindered by certain challenges: the target personnel in the unmanned end view in mountainous environments are usually small and in front of complex backgrounds. The proportion of small target size is less than 0.1% (such as pedestrians with 20 × 20 pixels), These types of targets lose information in deep networks due to multiple downsampling. The accuracy and robustness of small-target detection cannot meet the actual requirements. Deep learning methods used in target detection algorithms constitute a current research hotspot in unmanned airborne target detection owing to their capacity for high-precision feature extraction and real-time detection. The target detection model based on deep learning is mainly divided into single- and two-stage models according to the process. The two-stage model divides the detection process into two stages: generating candidate regions, followed by classification and bounding box regression on the candidate regions. Representative algorithms include the Region-Convolutional Neural Network [3] (R-CNN), Fast R-CNN [4], Faster R-CNN [5], and Mask R-CNN [6]. However, their two-stage detection processes lead to high computational complexity, slow inference, and numerous model parameters. These characteristics present two-stage detection algorithms with many challenges when deployed on UAV devices with limited arithmetic power. Single-phase target detection algorithms, such as RetinaNet [7], SSD [8], and the You Only Look Once (YOLO) series algorithms [9,10,11,12,13,14,15,16,17,18,19,20], are very suitable for deployment in real applications, especially in arithmetic devices such as UAVs.

To address the difficulty of small-target detection, in 2014, a Google research team introduced the Inception network architecture in GoogLeNet [21], the core idea of which is to improve the feature extraction ability of the model through multi-scale convolution and a parallel structure while controlling the amount of computation and the number of parameters. In 2018, Songtao Liu et al. [22] proposed the Receptive Field Block (RFB) module, which is designed to improve a model’s ability to detect multi-scale targets by simulating the receptive field mechanism of the human visual system. Through the introduction of the attention mechanism, the target detection model can capture the important regions and features in an image more efficiently, thus improving detection accuracy, especially in complex scenes (e.g., those involving weather and background interference). In 2018, Sanghyun Woo et al. [23] proposed the spatial attention mechanism, and Jie Hu et al. [24] proposed the channel attention mechanism. In 2022, Wang L. et al. [25] proposed an improved deep-learning-based SSD model that enhances small-target feature extraction in shallow networks by introducing an improved Inception network. In 2023, S. Tang et al. [26] proposed an improved YOLOv5 model called HIC-YOLOv5 that enhances small-target feature extraction in shallow networks by adding additional small-target-specific prediction heads, introducing involution blocks to enhance the channel information of the feature *mAP*, and applying the CBAM attention mechanism to improve small-target-detection performance. In 2024, Zhang M. et al. [27] designed an improved YOLOv8 [17] network specialized for detecting small targets in underwater images.

In recent years, there have been many small object detection and recognition algorithms based on YOLO, ranging from YOLOv7 to YOLOv11 [28,29,30,31,32,33,34,35]. With the continuous improvement of algorithms, the accuracy of detection and recognition has also increased. for example, Chen, J.G. et al. [30] conducted research on using improved YOLOv7 to achieve small object detection. They are effective for simple scenes such as wide fields, city roads, etc., but still perform poorly when applied to scenes with complex terrains, such as those common in mountain manhunts. As the number of network layers deepens and increases, under convolution and upsampling, the feature information pertaining to the target in the image inevitably reduces and is lost, resulting in the omission of small targets, in turn leading to a reduction in the detection accuracy of the final model.

To address these small-target detection difficulties, we designed a target detection network algorithm with multibranch convolution and attention combined with an improved C2F module to improve small-target detection accuracy for mountainous scenes from the viewpoint of a UAV.

## 2. Materials and Methods

### 2.1. Target Detection Network Based on Multi-Branch Convolution with an Attention-Improved C2F Module

**Figure 1:**
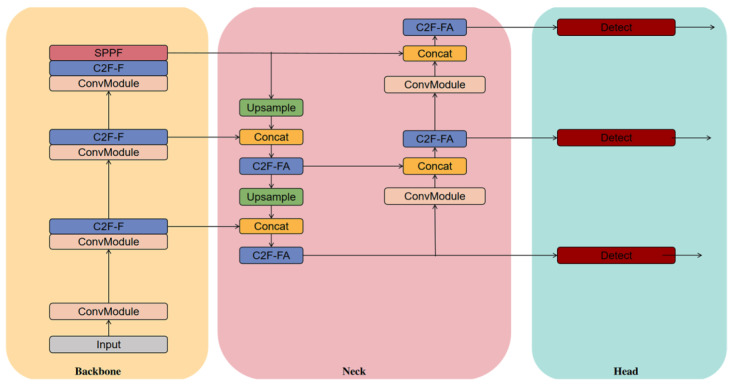
The structure of the target detection network based on multi-branch convolution and attention combined with an improved C2F module.

The structure of the target detection network based on multi-branch convolution with an attention improvement C2F module, shown in Figure 1, is divided into three parts. The first part is the backbone network, whose main function is to extract the input features. The second part is the neck network, which is responsible for fusing the features of different scales sent from the backbone network in layers to improve the characterization of the target features of different sizes. The third part is the detection head, which is responsible for outputting the class probabilities and bounding box coordinates of the objects of different scales.

### 2.2. Backbone Incorporating Partial Convolution

To cope with the limited arithmetic power of UAVs, the C2F module in the backbone network reference FasterNet network structure was improved to enhance the speed and efficiency of feature extraction in the backbone network.

The core idea of the FasterNet network [36] is to reduce the amount of computation through partial convolution (PConv) while ensuring feature extraction ability. Standard convolution, which is used in the C2F module in a traditional network, is a convolution operation performed on all channels of the input feature *mAP* to generate the output feature *mAP*. Each output channel is the weighted sum of all the channels of the input feature *mAP*, which performs the convolution operation on only some of the channels of the input feature *mAP*, while the other channels are directly passed to the next layer, thereby reducing the amount of computation while retaining some of the original feature information.

Assuming that the shape of the input network feature *mAP* is $\left(C_{in},H,W\right)$—which assumes the shape $\left(C_{out,}C_{in},K,K\right)$, where $C_{in}$ is the number of input channels, $C_{out}$ is the number of output channels, K is the size of the convolution kernel, and H and W are the height and width of the feature *mAP*, respectively—and the shape of the output feature *mAP* is $\left(C_{out},H^{\prime },W^{\prime }\right)$, where $H^{\prime }$ and $W^{\prime }$ are the height and width of the output feature *mAP*, PConv selects the first $C_{p}$ channel for the convolution operation, and the remaining $C_{in}-C_{p}$ channels are passed directly. The shape of the convolution kernel is $\left(C_{out,}C_{p},K,K\right)$. The ratio of the computational complexity of the standard convolution to that of PConv is as follows:

(1) $$\frac{O(C_{in}\times C_{out}\times K\times K\times H^{\prime }\times W^{\prime })}{O(C_{p}\times C_{out}\times K\times K\times H^{\prime }\times W^{\prime })},$$

**Figure 2:**
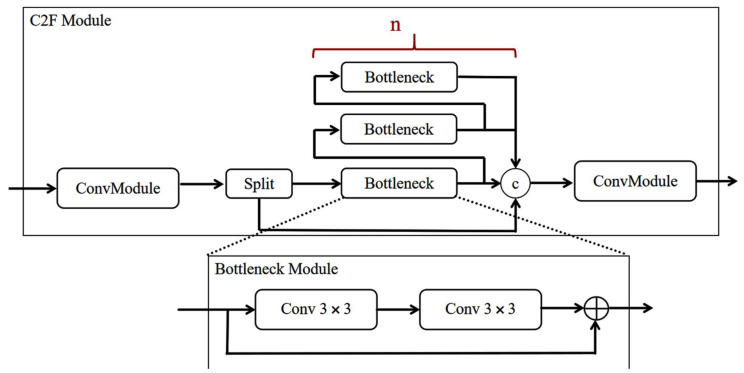
The structure of the C2F-F module.

Because $C_{p}<C_{in}$, network computation is significantly reduced. In this study, to improve the efficiency of the network, PConv was applied to the C2F modules to design the C2F-F module, whose structure is shown in Figure 2. The original standard convolution was changed to PConv, which utilizes the characteristics of PConv partial convolution to reduce complexity and improve network computation efficiency. Table 1 shows that after the C2F-F module was employed, the number of parameters reduced by 10.46%, and the required floating-point operations reduced by 0.6 GFLPs, with an increase in the number of network layers, proving that the module can reduce the number of network parameters and improve the efficiency of network computation.

### 2.3. FA-Block Based on the Design of a Multi-Scale Feature Fusion and Attention Mechanism

Aiming to address the neck network’s difficulty detecting small targets, we combined multiscale feature fusion with channel and spatial attention mechanisms in the neck network to design the FA-Block. This block expands the sensory field of the network and enhances its ability to extract small-target features through multiscale feature fusion and an attention mechanism. Meanwhile, we replaced the bottleneck structure in the C2F module with the FA-Block block and designed the C2F-FA block to replace the original C2F module to enhance feature fusion capacity and the ability to focus on small-target features.

**Figure 3:**
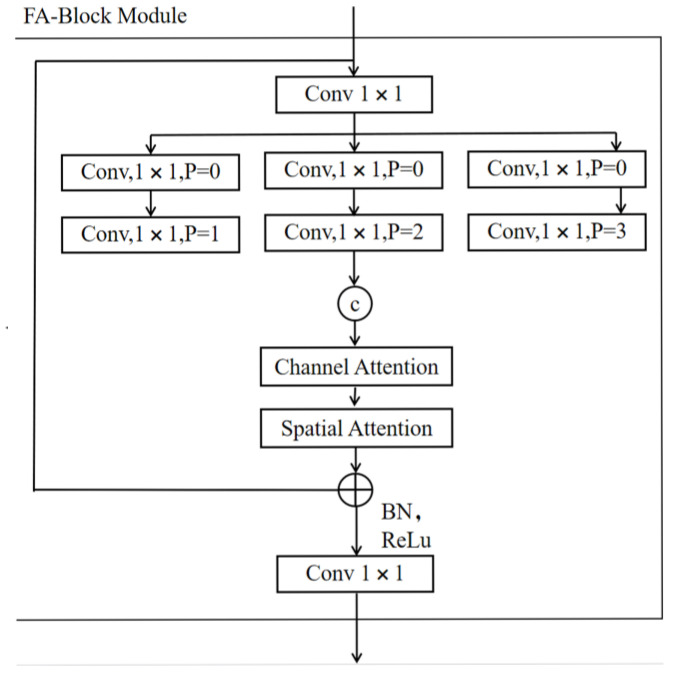
Structure of the FA-Block module.

The FA-Block block combines multibranch convolution with the attention mechanism to enhance the model’s feature fusion capacity and detection accuracy, as shown in Figure 3. The multibranch convolution component has three branches with dilation rates of 1, 2, and 3.

The base branch (d = 1) is a 5 × 5 standard convolution that captures local details, the middle-range branch (d = 2) is an equivalent 9 × 9 receptive field that extracts medium-range semantic information, and the remote branch (d = 3) is an equivalent 13 × 13 receptive field that models long-range spatial dependencies.

The output features of each branch were spliced through channels to form a multiscale feature tensor to help the network capture contextual information at different scales. Subsequently, the outputs of the three branches were spliced, and channel and spatial attention were applied.

Finally, the double-attention processed features were convolved by 1 × 1 to realize feature fusion and residual concatenation. This design is intended to enhance the diversity and robustness of the features, and it is hoped that this module will yield good results when dealing with targets at different scales. Output formula is

(2) $$\mathrm{Output}=Concat\left[Conv_{1\times 1}(x),Conv_{3\times 3}(x),Conv_{5\times 5}(x),MaxPool_{3\times 3}(x)\right],$$

**Figure 4:**
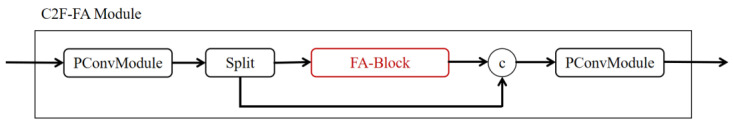
Structure of the C2F-FA module.

In this study, a C2F-FA module was designed by replacing the bottleneck blocks in the C2F module with FA-Blocks, as shown in Figure 4. Each bottleneck in the C2F structure was replaced with an FA-Block with an attention mechanism and multi-branch convolution, and the number was reduced to one, thereby enhancing the expressiveness of the model and balancing the network’s complexity throughout the feature extraction process.

### 2.4. Addition of a Tiny-Target Detection Layer

Small targets have fewer pixels, are easily overwhelmed by background targets and background noise, and exhibit weak feature expression; conventional down-sampling will result in the loss of small-target features or their confusion with background noise. In this study, we designed a tiny-target layer with the following dimensions: 160 × 160 × 128.

**Figure 5:**
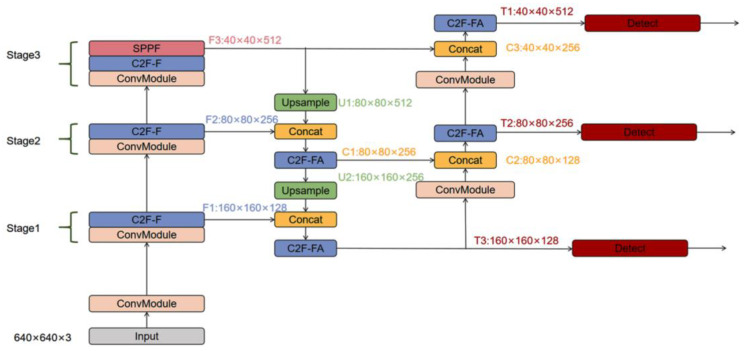
Change in the dimensions of the improved network feature *mAP*.

The change in the size of the feature *mAP* after the improvement is shown in Figure 5 and Table 2. T3 has fewer convolutions, and the feature *mAP* is larger, a state more conducive to small-target recognition. In this study, optimizing the network structure enabled an increase in the tiny-target layer, which focuses on extracting small-scale features, allowing the network to better meet the current requirements of UAV small-target detection.

When the dimensions of the input image are 640 × 640, the 160 × 160 layer corresponds to 4-fold down-sampling with the following number of pixels:

(3) $$\frac{640}{4}\times \frac{640}{4}=25,600$$

The number of pixels in the original 8-fold down-sampling procedure is as follows:

(4) $$\frac{640}{8}\times \frac{640}{8}=6400$$

A comparison of the formulas reveals that the improved tiny-target layer significantly increases the number of pixels for small targets.

### 2.5. Improvements to the Up-Sampling Methodology

**Figure 6:**
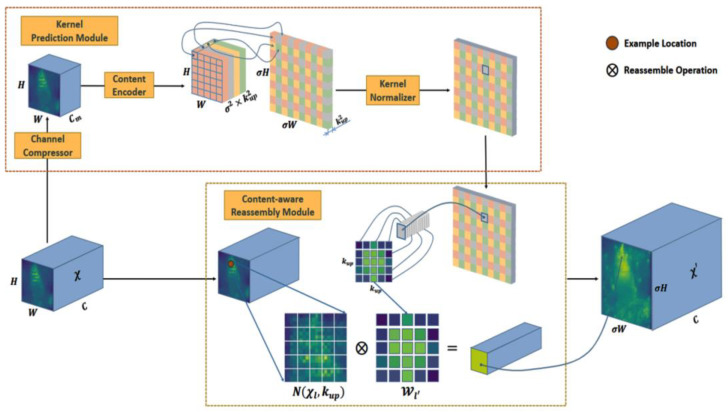
Structure of the CARAFE module.

To improve UAVs’ recognition performance with respect to small targets, we improved the neck of the base network and adopted the lightweight up-sampling operator CARAFE to replace the original nearest-neighbor interpolation up-sampling operator. As shown in Figure 6 [37], the CARAFE operator consists of an up-sampling kernel prediction module and a feature reorganization module.

There are two steps in the CARAFE module. The first step is predicting a reorganization kernel based on the content of each target location, and the second step is reorganizing the features with the predicted kernel.

In the up-sampling kernel prediction module, assuming that the up-sampling multiplicity of the network is σ and the shape of the output feature *mAP* X of the previous layer is H × W × C, the module compresses the number of channels of the feature *mAP* to $C_{m}$ via a 1 × 1 convolution operation and then predicts the up-sampling kernel with a convolutional layer of $k_{e}\times k_{e}$, resulting in an up-sampling kernel with the following shape: $\sigma H\times \sigma W\times k_{up}^{2}$. Finally, this up-sampling kernel is normalized using the Softmax pair in order to ensure that the weights of the convolution kernel sum to 1.

In the feature recombination module, region-centered $k_{up}\times k_{up}$ at each position of the output feature *mAP* is extracted, and the recombined kernel $W_{t}$ is subjected to dot-product computation to enhance the semantic information of the feature *mAP*.

The optimized shallow network focuses on capturing detailed information to accurately locate the target, whereas the deep network focuses more on understanding contextual semantic information for inference. Applying CARAFE to the neck network feature pyramid FPN can enhance the deep network’s characterization ability, thereby endowing the fused features with a richer expressive capacity.

This method was developed in combination with the special needs of mountainous scenes. Optimized the network structure for mountain small target recognition, improving the robustness of the model in special scenarios. Although there are some limitations in the design of the network structure, we have actively explored a network structure suitable for mountain unmanned aerial vehicle search missions.

## 3. Results

### 3.1. Dataset and Experimental Setup

In the experiments, we used the mountain pedestrian dataset, a target detection dataset consisting of images taken from a UAV’s viewpoint collected and produced in this study. The dataset contains two types of detection objects: people and occluded pedestrians. We used a total of 7464 datasets, and the images were randomly assigned in a ratio of 8:1:1 for the training, validation, and test sets.

### 3.2. Environmental Configuration and Evaluation Indicators

The parameter settings for the experimental platform are shown in Table 3.

Using the PyTorch 2.5.0 framework on an Ubuntu 20.04 server, we conducted the experiments using a server configuration consisting of an Intel(R) Xeon(R) Gold 6338 CPU operating @ 2.00 GHz (Intel, Santa Clara, CA, USA), 1 T of RAM, and four Nvidia RTX 4090 24 G GPUs (Nvidia, Santa Clara, CA, USA).

Environment configuration: Python version 3.8, torch version 2.4.1, CUDA version 12.1, and YOLO version 8.0.138.

The dimensions of each image were 640 × 640, the number of training rounds was 150, and the single-image input batch was set to 16. The remaining experimental parameters were set according to the system’s default settings. Precision (*P*), recall (*R*), and mean average precision (*mAP*) were used as experimental evaluation metrics, and two specific versions of *mAP*50 and *mAP*50-95 were used for mean precision. Equations (5), (6), (7), and (8) are the formulae for *P*, *R*, *mAP*50, and *mAP*50-95, respectively.

(5) P=TP/(TP+FP),

(6) R=TP/(TP+FN),

(7) $$mAP=\frac{1}{m}\sum_{i=1}^{m}AP_{i}^{IoU=0.5},$$

(8) $$mAP=\frac{1}{m}\sum_{i=1}^{m}AP_{i}^{IoU=0.5:0.05:0.95},$$

### 3.3. Ablation Experiment

To verify the performance impact each module had on the network, we performed ablation experiments using the mountain pedestrian dataset, using YOLOV8n as the base network.

Table 4 shows the performance impact of each module of the improved algorithm on the network with respect to the mountain pedestrian dataset. In comparison to the performance of the base network, each module improved the model. Still effective when the modules acted together, the improved network improved the *mAP*50, *mAP*50-95, *P*-value, and *R*-value by 2.8%, 3.5%, 2.3%, and 0.2%, respectively. The detection of small targets was further improved, demonstrating the effectiveness of the target detection network incorporating multi-branch convolution and attention combined with an improved C2F module.

To verify the generalization ability and robustness of each module of the improved network, we also used the public dataset VisDrone2019, as shown in Table 5. In comparison to the base network, the *mAP*50, *mAP*50-95, *P*-value, and *R*-value improved by 9.2%, 6.4%, 7.7%, and 7.6%, respectively. The experimental results show that even when applied to the public dataset VisDrone2019, the modules in the target detection network incorporating multibranch convolution and attention combined with the improved C2F module are still effective, demonstrating the robustness of the improved network algorithm.

### 3.4. Comparison of the Results for Different Algorithms

To verify the model’s performance, we compared it with several other mainstream target detection models, including the YOLOV5n, YOLOV5s, YOLOV7n, YOLOV8n, and YOLOv11n models, which are more advanced target detection models.

YOLOv11 is the latest iteration version of the YOLO model developed by the Ultralytics team. YOLOv11 has introduced architectural improvements to optimize feature extraction and processing on the solid foundation of YOLOv8, which has resulted in better performance of YOLOv11 in small object processing compared to YOLOv8. Therefore, this article also includes YOLOv11 in the comparison method.

Table 6 presents the quantitative results obtained with respect to the mountain pedestrian dataset. The *mAP*50, *mAP*50-95, *P*-value, and *R*-value of the improved algorithm model in this study were 85.5%, 46.1%, 80.9%, and 82.9%, respectively, constituting the best figures among all the models, and the side-by-side comparisons verify the effectiveness of the model designed in this study.

Table 7 shows the test results obtained for the various algorithms with respect to VisDrone2019. Compared with the best-performing algorithm model in regard to VisDrone2019, the *mAP*50, *mAP*50-95, *P*, and *R* values of the model designed in this study are 3.4%, 4.8%, 2%, and 0.9% better, respectively. These results prove that our model can effectively and accurately detect small target objects.

### 3.5. Visualization Results

**Figure 7:**
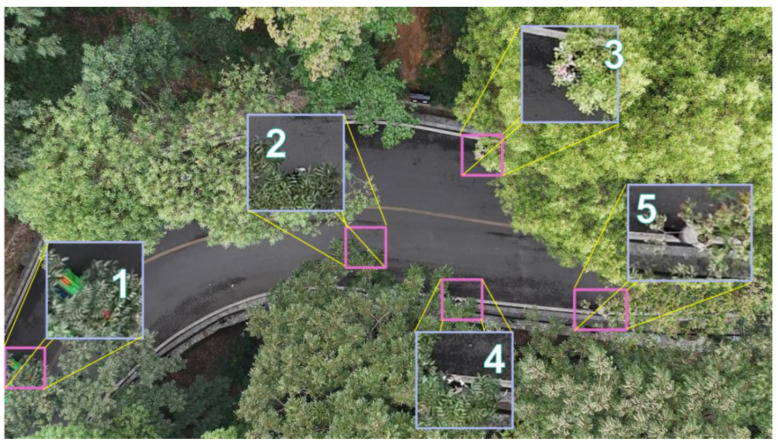
Enlarged view of the local details of the test pictures.

To more concretely prove the effectiveness of our improved C2F modular target detection network based on the combination of multi-branch convolution and attention for small-target detection, pictures were randomly selected from the mountain pedestrian dataset and used to test the improved algorithmic model and other mainstream detection algorithmic models, respectively. Local magnified details of the original image are shown in Figure 7, in which square 1 is a vehicle, which was not a detection target, whereas squares 2 to 5 are occluded pedestrian targets.

**Figure 8:**
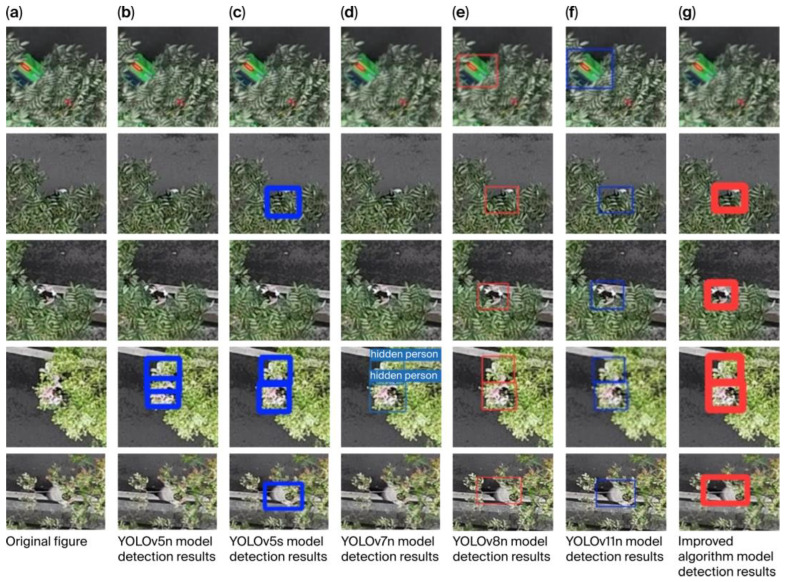
Comparison of the detection results for the original, YOLOv5n, YOLOv5s, YOLOv7n, YOLOv8n, YOLOv11n, and improved algorithmic models.

Figure 8 shows the results of comparing the detection effectiveness of the other algorithmic models with that of the improved algorithmic model. Column a is the original image, while columns b-g correspond to the detection results for the different models. The first row shows that the YOLOv8n and YOLOv11n models misrecognized vehicles as occluded pedestrians; the second, third, and fifth rows show that the YOLOv5n, YOLOv5s, and YOLOv7n models failed to detect occluded people. In contrast, the improved model accurately recognized all occluded pedestrians and did not misidentify the vehicle as an occluded person, demonstrating its superior reasoning ability for small targets and higher detection accuracy relative to the other algorithmic models, comprehensively proving its effectiveness in small-target detection.

## 4. Conclusions

In this paper, we address the current problems in UAV detection attributable to the limited arithmetic ability of UAVs and the inability of mainstream detection algorithms to effectively deal with small targets. Referring to the partial convolutional (PConv) layers in the FasterNet network in the backbone network, we designed the C2F-F module to utilize the characteristics of partial convolutions to improve the network’s computational efficiency. In the neck network, the FasterNet Block was improved by combining multi-scale feature fusion with channel and spatial attention mechanisms, and the FA-Block block was designed. The bottleneck structure in the C2F block was replaced with the FA-Block, and the C2F-FA block was designed to replace the original C2F block to improve the network’s feature fusion capacity and ability to focus on small-target features. A tiny 160 × 160 target layer was added to improve the extraction of small-scale features, thus enabling the network to better meet the current requirements of UAV small-target detection. Finally, CARAFE was applied to the neck network feature pyramid FPN to enhance the network’s deep characterization capacity, thus endowing the fused features with richer expressive capabilities.

In the ablation experiments, the target detection network designed in this study based on multi-branch convolution and attention combined with the improved C2F module exhibited improved experimental metrics in comparison to the original network on the self-built dataset and the publicly available dataset VisDrone dataset, proving its effectiveness in detecting small targets. In the experiments comparing five mainstream detection algorithms on different datasets, all the metrics for our algorithm were superior to those of the other algorithms. In the visualization comparison of the detection results, the improved algorithm model accurately identified all the obscured pedestrians and did not misidentify the vehicles as obscured people, showing its superior reasoning ability and detection accuracy for small targets relative to the other models, concretely proving its effectiveness in the field of small-target detection. This study reveals the effectiveness of the model designed in this study and its good robustness.

In addition to the influence of terrain, adverse weather conditions also have a significant impact on the recognition of small targets, which requires the use of multiple sensors and multi-source image fusion to solve. This is the next research direction.

## Figures and Tables

**Table 1:** A comparison of the structures and computational complexity of the models.

	Layers	Parameters	GFLOPs	FPS
Base	225	3,157,200	8.9	11
C2F-F	231	2,826,960	8.3	8.72

**Table 2:** Change in the size of the improved network feature *mAP*.

Network Stage	Name of Feature *mAP*	Dimensions (W × H × Aisle)	Operation Description
Backbone	Input	640 × 640 × 3	Original input
Backbone	F1	160 × 160 × 128	Generated via convolution operation on input
Backbone	F2	80 × 80 × 256	Generated via F1 convolution operation
Backbone	F3	40 × 40 × 512	Generated via F2 convolution operation
Neck	U1	80 × 80 × 512	F3 is up-sampled
Neck	C1	80 × 80 × 256	Generated by concatenating U1 and F2
Neck	U2	160 × 160 × 256	C1 is up-sampled
Head	T3	160 × 160 × 128	U2 is spliced with F1 and fed into the detector head
Head	T2	80 × 80 × 256	C1 is spliced with T3 and then generated and fed into the detection head
Head	T1	40 × 40 × 512	F3 and T2 are spliced, generated, and fed into the detector head

**Table 3:** Configuration of the experimental environment.

Project	Version
Operating System	Ubuntu 20.04
CPU	Intel Xeon Gold 6338
GPU	Nvidia RTX 4090 24 G
Compiler	PyCharm
Algorithmic Framework	Pytorch-2.4.1 + Cuda12.1
Programming Languages	Python3.8
YOLO Version	v8.0.138

**Table 4:** The performance impact of each module on the network. ‘—’ Represents that this module has not been added,‘√’ Represents that this module has been added.

C2F-F	C2F-FA	Tiny	CARAFE	*mAP*50/%	*mAP*50-95/%	*P*/%	*R*/%
—	—	—	—	82.7	42.6	78.6	82.7
√	—	—	—	82.8	43.0	78.7	80.6
—	√	—	—	83.1	43.2	78.3	80.1
—	—	√	—	84.5	45.0	79.6	81.4
—	—	—	√	83.0	42.9	79.0	80.4
√	√	√	√	**85.5**	**46.1**	**80.9**	**82.9**

**Table 5:** The effectiveness of each module with respect to the VisDrone2019 dataset. ‘—’ Represents that this module has not been added,‘√’ Represents that this module has been added.

C2F-F	C2F-FA	Tiny	CARAFE	*mAP*50/%	*mAP*50-95/%	*P*/%	*R*/%
—	—	—	—	32.1	18.3	43.2	32.6
√	—	—	—	32.3	18.7	42.7	32.3
—	√	—	—	38.2	22.6	49.6	37.3
—	—	√	—	35.2	20.6	44.5	35.5
—	—	—	√	33.0	19.0	42.5	33.4
√	√	√	√	**41.3**	**24.7**	**50.9**	**40.2**

**Table 6:** The test results for the different algorithms when applied to the self-constructed dataset.

Model	*mAP*50/%	*mAP*50-95/%	*P*/%	*R*/%
YOLOv5n	77.1	34.5	75.7	75.0
YOLOv5s	82.1	40.0	78.9	79.1
YOLOv7n	72.8	32.1	71.3	73.2
YOLOv8n	82.7	42.6	78.6	82.7
YOLOv11n	82.3	42.1	77.7	80.1
**Ours**	**85.5**	**46.1**	**80.9**	**82.9**

**Table 7:** Test results obtained for the different algorithms on the VisDrone2019 dataset.

Model	*mAP*50/%	*mAP*50-95/%	*P*/%	*R*/%
YOLOv5n	25.3	13.0	35.5	27.7
YOLOv5s	32.9	18.2	44.5	33.2
YOLOv7n	37.9	19.9	48.9	39.3
YOLOv8n	32.1	18.3	43.2	32.6
YOLOv11n	34.2	19.9	45.1	33.8
**Ours**	**41.3**	**24.7**	**50.9**	**40.2**

## Data Availability

The data supporting this article have been uploaded at https://pan.baidu.com/s/1X5b7qtDRcDnk2nyw2rXoRg (accessed on 23 September 2025). The extraction code is hv33. links of the Code: https://github.com/hunnutangweiwei/-multi-branch-convolution-and-attention-improved-C2F-module (accessed on 23 September 2025).
